# The ryanodine receptor leak: how a tattered receptor plunges the failing heart into crisis

**DOI:** 10.1007/s10741-012-9339-6

**Published:** 2012-08-30

**Authors:** Thomas H. Fischer, Lars S. Maier, Samuel Sossalla

**Affiliations:** Department of Cardiology and Pneumology, Heart Center, Georg-August-University Göttingen, Robert-Koch-Str. 40, 37075 Göttingen, Germany

**Keywords:** Heart failure, Ryanodine receptor, SR Ca^2+^ leak, PKA, CaMKII

## Abstract

It has been persuasively shown in the last two decades that the development of heart failure is closely linked to distinct alterations in Ca^2+^ cycling. A crucial point in this respect is an increased spontaneous release of Ca^2+^ out of the sarcoplasmic reticulum during diastole via ryanodine receptors type 2 (RyR2). The consequence is a compromised sarcoplasmic reticulum Ca^2+^ storage capacity, which impairs systolic contractility and possibly diastolic cardiac function due to Ca^2+^ overload. Additionally, leaky RyR2 are more and more regarded to potently induce proarrhythmic triggers. Elimination of spontaneously released Ca^2+^ via RyR2 in diastole can cause a transient sarcolemmal inward current and hence delayed after depolarisations as substrate for cardiac arrhythmias. In this article, the pathological role and consequences of the SR Ca^2+^-leak and its regulation are reviewed with a main focus on protein kinase A and Ca^2+^-calmodulin-dependent kinase II. We summarise clinical consequences of “leaky RyR2” as well as possible therapeutic strategies in order to correct RyR2 dysfunction and discuss the significance of the available data.

## Prevalence and socioeconomic relevance of heart failure

Heart failure is characterised by a progressive deterioration of cardiac function and represents a major public health burden. Despite a number of well-established therapies, the prevalence as well as the mortality of heart failure are still alarmingly high. Heart failure remains the most frequent reason for hospital admission of patients older than 65 years [1]. In the USA, the number of hospitalisations due to heart failure has more than tripled between 1979 and 2004 [2]. The over-all prevalence of heart failure is estimated to be about 2 % [3]. More than 10 % of people older than 80 years are affected, many of them are highly limited as to their physical capacities and thus do suffer significantly in everyday life. Furthermore, the prognosis of heart failure patients is still miserable. There are estimated survival rates of only 50 % 5 years and 10 % 10 years after diagnosis [4–6].

## Aetiology and manifestations of heart failure

Huge efforts have been made to further elucidate underlying pathomechanisms and identify new targets for innovative therapies. The aetiologies of heart failure are various, stretching from coronary artery disease and heart valve dysfunctions to toxic influences on the myocardium or infectious diseases. Arterial hypertension could be identified as the most frequent risk factor in this context [7]. The common final path is an impairment of myocardial function. Importantly, systolic as well as diastolic function can be compromised independently leading to similar clinical manifestations. Current definitions of heart failure have taken this into account by distinguishing between heart failure with preserved ejection fraction (HFpEF) and heart failure with reduced ejection fraction (HFrEF). According to the guidelines of the American Heart Association, HFpEF is defined by symptomatic heart failure and a left ventricular ejection fraction of at least 50 % [8]. This entity has gained increasing attention in recent years as up to 50 % of heart failure patients can indeed be classified there [1, 9] and evidence-based medical therapy is still lacking.

On a histological and cellular level, heart failure could be linked to several alterations including fibrosis, inflammation and apoptosis. Furthermore, it has become more and more obvious that a fully functional excitation–contraction coupling process in the cardiomyocyte is fundamental for both, a normal systolic and diastolic function.

## Excitation–contraction coupling and its regulation

**Fig. 1:**
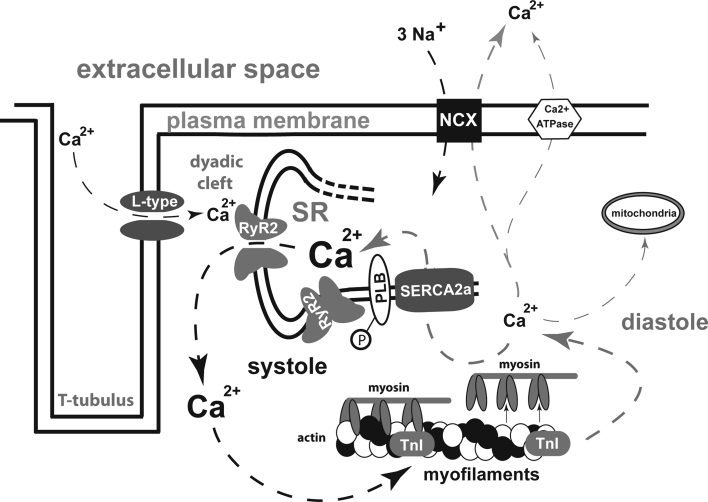
Mechanisms of excitation–contraction coupling in cardiomyocytes; *arrows* indicate Ca^2+^ shifts in systole (*left*) and diastole (*right*); *L*-*type* L-type Ca^2+^ channel; *RyR2* ryanodine receptor type 2; *SR* sarcoplasmic reticulum; *PLB* phospholamban; *TnI* troponin I; *NCX* Na^+^–Ca^2+^-exchanger; *SERCA2a* sarcoplasmic endoplasmic reticulum Ca^2+^-ATPase 2a; *P* phosphate

Physiological excitation–contraction coupling is the basis of a sound periodic succession of contraction and relaxation. It is characterised by a well-defined, triggered Ca^2+^ release out of the sarcoplasmic reticulum (SR) in systole followed by a quick Ca^2+^ elimination out of the cytosol to induce relaxation in diastole (Fig. 1).

During phase 0 of an action potential fast Na^+^-influx leads to a steep increase of the membrane potential increasing the probability that the voltage dependent L-type Ca^2+^ channels will open. Thereupon Ca^2+^-ions passively drawn by a concentration and electrochemical gradient enter the cytoplasm and maintain the plateau phase of the action potential. Inside the cell, they bind to ryanodine receptors type 2 (RyR2) that are located on the surface of the SR and trigger the release of an even bigger amount of Ca^2+^ out of the SR. This mechanism of Ca^2+^-induced Ca^2+^ release leads to a prominent increase in cytosolic Ca^2+^ that binds to troponin C (TnC) and thereby induces actin–myosin interaction. In diastole, Ca^2+^ has to be eliminated from the cytosol to allow relaxation of the myofilaments. This is mainly achieved by an active, energy-consuming Ca^2+^-reuptake into the SR via the sarcoplasmic reticulum Ca^2+^-ATPase type 2a (SERCA2a). A smaller amount of Ca^2+^ is extruded out of the cell via the Na^+^/Ca^2+^-exchanger (NCX), which is a facilitated diffusion in which the electrochemical potential gradients of Na^+^ and Ca^2+^ are the source of energy to drive the transport. NCX in its “forward” mode produces an electrical current because 3 Na^+^ are exchanged for 1 Ca^2+^. In human cardiomyocytes, the reuptake of Ca^2+^ into the SR makes up for approximately 70 % of the systolic Ca^2+^ depending on the heart rate [10]. Around 28 % are pumped out of the cell via NCX. The remaining 2 % are allotted to Ca^2+^ uptake into mitochondria and elimination via Ca^2+^-ATPases in the plasma membrane [11].

As the cyclic alterations of cytosolic Ca^2+^ concentrations make up the molecular trigger for cardiac contraction and relaxation, it is a matter of course that this system is elaborately regulated and highly adaptable to physical demands. SERCA2a activity is influenced by phospholamban (PLB) that is one of the major mediators of the cardiac contractility response upon β-adrenergic stimulation [12]. Inhibition of SERCA2a by PLB is dependent on the phosphorylation status of PLB and is most pronounced when PLB is unphosphorylated. Protein kinase A (PKA) as well as the Ca^2+^/calmodulin-dependent protein kinase IIδ (CaMKIIδ) is able to phosphorylate PLB at specific residues (serine 16 and threonine 17, respectively) and thereby abandon its inhibitory effect on SERCA2a. A second subcellular microdomain is the RyR2-complex that consists of several proteins and whose Ca^2+^ release capacity and diastolic closure can be regulated via phosphorylation by PKA and CaMKII [13–19] at Ser2809 and Ser2815, respectively. Furthermore, protein phosphatases 1 and 2a were found to regulate the phosphorylation status of several Ca^2+^ handling proteins [20].

## Consequences of altered excitation–contraction coupling in heart failure: the diastolic leak

**Fig. 2:**
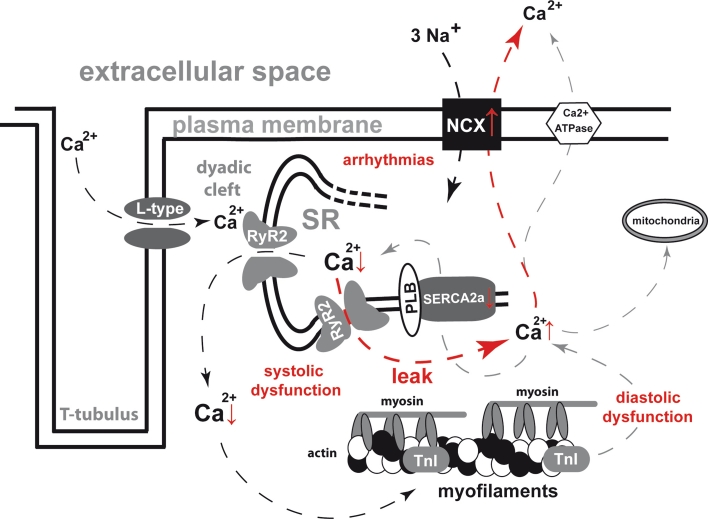
Deteriorations of excitation–contraction coupling in heart failure; *arrows* indicate Ca^2+^ shifts in systole (*left*) and diastole (*right*); diastolic Ca^2+^ leak is *highlighted* in *red*; changes in protein function and ion concentrations are indicated by *vertical red arrows*; *↑* increase; *↓* decrease; *L*-*type* L-type Ca^2+^ channel; *RyR2* ryanodine receptor type 2; *SR* sarcoplasmic reticulum; *PLB* phospholamban; *TnI* troponin I; *NCX* Na^+^–Ca^2+^-exchanger; *SERCA2a* sarcoplasmic endoplasmic reticulum Ca^2+^-ATPase 2a; *P* phosphate

Several animal models as well as functional analyses of human tissue samples have persuasively shown that Ca^2+^ cycling is profoundly disturbed in heart failure (Fig. 2). The most distinctive aspect is a reduction of systolic Ca^2+^ transient amplitude, which is commonly accompanied by an increase of diastolic Ca^2+^ levels in the cytosol. Furthermore, the velocity of diastolic Ca^2+^ elimination is reduced [21], and the physiologically positive force-frequency relation is typically blunted and rather negative-shaped [10, 22]. These alterations resulting from the Ca^2+^ depletion of the SR in heart failure can be attributed to two distinct pathomechanisms with synergistic effects on SR Ca^2+^ load. On the one hand, the diastolic refill of the SR is compromised due to a decreased expression and activity of SERCA2a [10, 23, 24] and a reduction of PLB phosphorylation [25]. The regulation of the PLB-SERCA2a-interaction and the resulting new therapeutic options for the treatment of heart failure have recently been comprehensively reviewed [26].

On the other hand, there is an increased diastolic Ca^2+^ loss out of the SR via substantially altered RyR2. It has been widely shown in different animal models as well as in human tissue that Ca^2+^ can leave the SR during diastole via numerous short and tightly localised Ca^2+^ release events, the so-called Ca^2+^ sparks. A Ca^2+^ spark is generated from a limited number of clustered RyR2s that form a Ca^2+^ release unit. The spontaneous opening of one receptor can consecutively trigger the activation of neighbouring RyR2s of the same Ca^2+^ release unit via Ca^2+^-induced Ca^2+^ release (CICR) in the dyadic cleft. This locally restricted Ca^2+^ release is accompanied by a local decrease of SR Ca^2+^ content in the corresponding areas, the so-called Ca^2+^ blinks [27], and was shown to terminate when the local SR Ca^2+^ content reaches a threshold of approximately 60 % of the diastolic SR Ca^2+^ concentration [28]. The stability of RyR2-closure is severely compromised in heart failure leading to an increased diastolic open probability and thus an increased diastolic leak. Furthermore, the Ca^2+^ ions, once released, are partly removed from the cytosol via the NCX, which was found to be overexpressed in heart failure [10, 24, 29]. Extruding two positive electric charges (1 Ca^2+^ ion) in exchange for three (3 Na^+^ ions) leads to a depolarisation of the cell membrane and can trigger delayed after depolarisations [30]. Thus, as simple as the physiological task of the RyR2 complex to open quickly and widely in systole and to close tightly and densely in diastole might appear as far-reaching are the cellular consequences once it is disturbed. A leaky RyR2 could possibly be linked to all major drawbacks of heart failure: impaired systolic force generation, compromised passive diastolic relaxation and a disposition for arrhythmias. Therefore, huge efforts have been made in the last two decades to further elucidate mechanisms of RyR2 regulation in different physiological states as well as in different cardiac pathologies [13–15, 17, 18, 27, 31–37].

## The ryanodine receptor type 2: the gatekeeper of Ca^2+^ storage

**Fig. 3:**
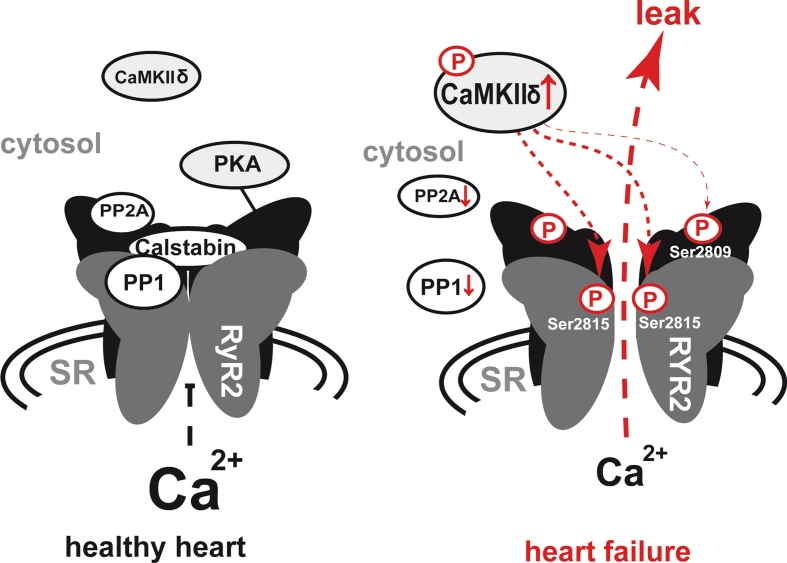
Development of an increased diastolic Ca^2+^ leak in heart failure; normal diastolic ryanodine receptor 2 (RyR2) closure (*left*) vs. diastolic SR Ca^2+^ leak due to spontaneous opening of a hyperphosphorylated RyR2 complex (*right*); the four subunits of the RyR2 complex are illustrated in *grey* and *black*; the diastolic leak is *highlighted* in *red*; relevant phosphorylation sites at the RyR2-protein are indicated; changes in protein function are indicated by *vertical red arrows*; *↑* increase; *↓* decrease; *SR* sarcoplasmic reticulum; *PP1* protein phosphatase 1; *PP2A* protein phosphatase 2A; *CaMKIIδ* Ca^2+^ calmodulin depending kinase IIδ; *PKA* protein kinase A; *P* phosphate

Ryanodine receptors are the largest known ion channels. In the heart, ryanodine receptor type 2 is the predominantly expressed isoform. It is a huge macromolecular protein exceeding a molecular weight of 2 MDa. Structural analysis have shown that RyRs are mushroom-shaped assemblies of four identical subunits each comprising approximately 5,000 amino acids [38]. This core part of the RyR2-complex then serves as a molecular scaffold for further proteins that attach to the receptor and regulate its function. Calsequestrin, junctin, triadin and FK506-binding protein, 12.6 kDa, (FKBP12.6) were shown to associate with the RyR2 complex [39–42] (Fig. 3). Calsequestrin is linked to the RyR2 complex via junctin and triadin [43] and acts as a Ca^2+^ buffer thus influencing RyR2 behaviour. Furthermore, RyR2 function is crucially linked to its phosphorylation status, which can be seen as the net effect of a steady competition between two major cellular kinases, PKA and CaMKII, and two protein phosphatases, PP1 and PP2a, all of which are closely associated with the receptor [44]. This close proximity enables dynamic changes of the phosphorylation status upon subcellular signals [44]. The relative phosphorylation of the receptor complex influences its Ca^2+^ sensitivity and thus the diastolic open probability as well as the systolic Ca^2+^ release capacity [14, 45] by inducing conformational changes of the RyR2 complex. The RyR2 complex therefore acts as a molecular switchboard, transducing cellular signals to the Ca^2+^ release pore and thus adapting Ca^2+^ release kinetics to cellular demands.

## Mechanisms of RyR2 dysregulation in heart failure

The mechanisms of RyR2 regulation have been subject of intense research in the last years due to their pathologic relevance. It has become undoubtedly clear that phosphorylation of the receptor protein is a key event in this context. The RyR2 complex thereby was found to be one of the first proteins that undergo phosphorylation upon beta-adrenergic stimulation [19]. As early as in 1991, it was described that RyR2 can be phosphorylated by CaMKII in canine cardiomyocytes, which leads to an activation of this Ca^2+^-release channel [19]. Furthermore, it could be demonstrated shortly after by a different group that the phosphorylation of RyR2 receptors in rat cardiomyocytes upon beta-adrenergic stimulation was PKA dependent [46]. From that time on, both kinases were intensively studied as to their impact on the RyR2 in different animal models as well as human heart tissue—with controversial results up to date.

## The role of PKA in the development of an increased SR Ca^2+^-leak

It was described by Marks and colleagues that PKA phosphorylation of the RyR2 leads to a dissociation of FKBP12.6 from the channel and consecutively to an increased Ca^2+^ sensitivity and compromised diastolic RyR2 closure [14]. Some years later, the same group proposed Ser2808 as the only functional PKA phosphorylation site on the RyR2 by showing that immunoprecipitated RyR2 from S2808A mutant mice could not be phosphorylated by PKA [16]. In contrast to their wild-type littermates these S2808A mutant mice did not show any PKA phosphorylation of RyR2 after isoproterenol treatment in vivo. Furthermore, these mice exhibited significantly improved cardiac function compared to wild type after myocardial infarction and were thus protected from maladaptive remodelling. Marks and colleagues also found a hyperphosphorylation of RyR2 at S2809 in overt human heart failure [16]. On account of these findings, a hyperphosphorylation of RyR2 by PKA had been proposed as central mechanism in the pathogenesis of heart failure. The scenario of persistent PKA activation in heart failure due to chronic activation of the sympathetic nervous system also fits to clinical observations, as most heart failure patients do have elevated catecholamine levels. Beta-adrenergic receptors have been reported to be downregulated [47]. Other groups, however, contradicted the data on the functional relevance of PKA-dependent RyR2 hyperphosphorylation. It could be shown in mouse ventricular myocytes that PKA phosphorylation of the RyR2 did not significantly affect diastolic SR Ca^2+^ leak [48]. Furthermore, another group rather found an intact beta-adrenergic response and an unmodified progression towards heart failure in mice with the same genetic ablation of the major PKA phosphorylation site (S2808A) [49]. In this study, the authors only found slight modifications of single-channel activity in Ser2808-mutated mice that allowed for normal Ca^2+^ transients and unaltered overall cellular function. Obvious protection against maladaptive remodelling after TAC was absent. Moreover, they did not find a significant impact of RyR-S2808A mutation on the diastolic SR Ca^2+^ leak and showed that RyR2-S2808A mutants could still be phosphorylated by PKA, which might be attributed to another phosphorylation site at S2830. The authors further concluded that genetic ablation of one single phosphorylation site might lead to conformational changes and shift kinase activity to other, physiologically not equally relevant sites that in turn make up for the missing phosphorylation site. Furthermore, different amounts of phosphatases included in the isolated RyR2 complexes could, at least partially, explain the contradictory results of different works in the field. Another group just recently exposed RyR-S2808A-mutated mice to myocardial infarction. They, however, could not find any differences compared with wild-type littermates, neither as to global cardiac function and calcium cycling parameters after myocardial infarction nor as to infarct size [50].

## FKBP12.6: a key player of RyR stabilisation or just a side show

Several studies strongly support the hypothesis that phosphorylation of RyR2 at Ser2808 by PKA causes the RyR2-associated protein FKBP12.6 to dissociate from the receptor with the result of an increased propensity for diastolic SR Ca^2+^ release [14, 51, 52]. However, these observations were challenged by several groups. Xiao et al. [53] examined the binding behaviour of FKBP12.6 with unphosphorylated and phosphorylated RyR2 proteins and did not find significant differences. Complete phosphorylation of Ser2808 by exogenous PKA did not disrupt FKBP12.6-RyR2 complexes and a S2808D mutated receptor that corresponds to a constitutively phosphorylated state of Ser2808 and thus mimics a constitutive phosphorylation also retained the ability to bind FKBP12.6 [53]. Another group examined the effect of a sticky FKBP12.6 mutant on the development of heart failure [54]. They generated mice expressing a FKBP12.6 (D37S) mutant that binds with greater stability to the RyR2. These mice showed increased SR Ca^2+^ load but did not exhibit any attenuation of cardiac functional decline after aortic banding. Another aspect that argues against an important, physiologically relevant FKBP12.6-mediated RyR2 regulation is the small amount of FKBP12.6 normally existing in rat and mouse myocytes [55]. FKBP12.6 was found to be numerically far less abundant in these cells than RyR2-receptors. This in turn means that the bulk of RyR2 proteins physiologically already lacks FKBP12.6 and might explain why some groups did not see any obvious effect of physiologically expressed FKBP12.6 on RyR2 gating while studies using animals overexpressing FKBP12.6 showed relevant effects an SR Ca^2+^ leak and SR Ca^2+^ content [56, 57].

Whereas the activating effect of PKA on L-type Ca^2+^ channels [58] and SERCA2a via PLB phosphorylation [59] is indisputable, its direct impact on RyR2 function and the role of FKBP12.6 dissociation is still not finally elucidated. The discrepancies of different studies in this field are difficult to explain. The varying outcomes in different mouse models harbouring the same RyRS2808A mutation might, at least partially, be attributed to different genetic backgrounds of the mouse lines used. Furthermore, the potency of cardiomyocytes to compensate artificially introduced defects of Ca^2+^ cycling cannot be overestimated. It has been shown that mice with a conditional knockout of the NCX could escape death by an adaptive reduction of Ca^2+^ influx through L-type Ca^2+^ channels instead of upregulation of Ca^2+^ efflux mechanisms [60]. This, however, means in turn that the validity of data from genetically altered animal models and their applicability for the explanation of pathomechanisms in human disease have to be handled with care. Eventually, functional data in human tissue will be needed to resolve this issue.

## The role of CaMKII for the development of an increased SR Ca^2+^ leak

There is an increasing body of knowledge concerning the crucial role of CaMKII for modulation of RyR2 function. Different sequence analysis revealed that there are up to four serine and two threonine residues at the RyR2 that can be phosphorylated by CaMKII. The first report was published by Witcher et al. [19], showing a direct phosphorylation of the myocardial RyR2 at Ser2809 by CaMKII. However, site-directed mutagenesis experiments showed that CaMKII binding at RyR2 might not be at Ser2809, which is supposed to be the PKA-dependent phosphorylation site, but at Ser2815 [13]. CaMKII-dependent RyR2 phosphorylation thereby resulted in a more pronounced RyR2 activation than PKA-dependent RyR2 phosphorylation. Additionally, this group clearly showed that CaMKII-dependent RyR2 phosphorylation increased the open probability of the cardiac RyR2 in lipid bilayer experiments. Some years later, it was also shown by Huke et al. [36] that the phosphorylation of RyR2 at Ser2814 by CaMKII rather than PKA-dependent phosphorylation at Ser2808 is crucial for RyR2 function. However, there are also reports that suggested a reduced SR Ca^2+^ leak due to CaMKII phosphorylation of the cardiac RyR2 [61].

After the discovery of RyR2 phosphorylation by CaMKII, several other groups confirmed the pathologically relevant impact of CaMKII on RyR2 function in animal models as well as in human disease [15, 17, 18, 31, 34, 62]. A transgenic mouse line overexpressing the CaMKIIδc exhibited severe alterations of Ca^2+^ cycling and dilated heart failure [15, 34]. An increased diastolic Ca^2+^ leak in these mice lead to a severe depletion of SR Ca^2+^ storage and reduced systolic Ca^2+^ transients [15, 34].

Furthermore, Ai et al. [32] could show in 2005 that the enhanced SR Ca^2+^ leak in isolated cardiomyocytes from a rabbit heart failure model could be reduced by CaMKII inhibition but not by PKA inhibition. Curran et al. [63] proposed that even the inotropic response upon beta-adrenergic receptor stimulation is caused by CaMKII. The fact that CaMKII was shown to be activated by autophosphorylation as well as oxidation [64, 65] enables a vicious circle to set in during conditions of diastolic Ca^2+^ overload and shortage of energy supply in heart failure leading to chronic CaMKII activation. These findings could recently be confirmed for human heart failure by our group [17]. CaMKII expression and phosphorylation were shown to be increased in left and right ventricular tissue probes of human heart failure. Inhibition of CaMKII yielded a reduction of SR Ca^2+^ leak in human cardiomyocytes isolated from failing hearts and improved SR Ca^2+^ storage. Furthermore, positive inotropic effects could be detected in twitching muscle preparations after CaMKII inhibition during increasing stimulation frequencies. These data provide compelling evidence for an important pathological role of CaMKII in heart failure.

There is also increasing evidence for the role of the CaMKII-induced diastolic Ca^2+^ leak in the development of arrhythmias. Isolated cardiomyocytes from mice overexpressing CaMKIIδc [15] showed an increased incidence of early after-depolarisations compared with wild type leading to an increased number of potentially arrhythmic spontaneous action potentials [18]. These proarrhythmic events could be further increased by isoproterenol treatment and were significantly reduced by CaMKII inhibition [18]. A recent study could link the increased incidence of arrhythmias in ischaemic canine heart tissue to a CaMKII-related shortening of the Ca^2+^ signalling refractoriness [66]. It could further be shown that the progression of human heart failure is associated with a continuous increase in RyR2-mediated Ca^2+^ leak, which is paralleled by an increased incidence of diastolic spontaneous Ca^2+^ waves [33]. The aetiology underlying the development of heart failure might also play an important role regarding the cellular pathomechanisms involved. A recent study showed that RyR2 is only hyperphosphorylated at the CaMKII-dependent site Ser2815 in non-ischaemic, but not in ischaemic heart failure [67]. Accordingly, mice harbouring a mutation at Ser2814 (S2814A) were protected from afterload-induced heart failure after trans-aortic constriction but not from ischaemic heart failure after myocardial infarction. More studies will be needed to resolve this issue. Other studies directly linked the SR Ca^2+^ leak and an increased CaMKII activity to atrial fibrillation (AF), the most common sustained cardiac arrhythmia [68, 69]. It could be shown that CaMKII expression and its phosphorylation are significantly increased in right atria of AF patients compared with patients in stable sinus rhythm, which was accompanied by an increased RyR2 phosphorylation at the CaMKII-specific site Ser2814 [68, 69]. Diastolic Ca^2+^ levels were found to be increased as the result of an increased diastolic Ca^2+^ leak. Both findings could be normalised by CaMKII inhibition [68]. These data could be nicely confirmed by a recent study [70], which also showed that the SR Ca^2+^ leak could be reduced in AF cells by CaMKII inhibition whereas PKA inhibition did not yield relevant effects on the SR Ca^2+^ leak again stressing the role of CaMKII under pathophysiological conditions as compared to PKA.

## RyR2 stabilisers and CaMKII inhibitors

The use of CaMKII inhibitors in patients is significantly hindered by the ubiquitous expression of this enzyme in different organs. Currently available CaMKII inhibitors cannot be administered to patients due to unspecific effects and pharmacodynamic problems (e.g. oral application of peptide inhibitors). Major hurdles as to organ-specific attribution still have to be overcome. Gene therapy-based approaches might, however, be feasible in the near future. Additionally, several compounds directly leading to a stabilisation of diastolic RyR2 closure have already been developed. The major challenge thereby is to identify compounds that exert a beneficial effect on the diastolic leak without compromising systolic RyR2 opening and Ca^2+^ release. One of the first compounds that combined both features was JTV519. It was shown to improve diastolic and systolic function in isolated human failing myocardium but also had unspecific effects on other ion channels and a narrow therapeutic range with negative inotropic effects at higher concentrations [71–73]. A newer and more specific drug called S44121 has been developed and is currently being evaluated in a phase 2 multicentre clinical study (ISRCTN reg. number 14227980).

## Conclusion

Taken together, the RyR2-dependent SR Ca^2+^ leak has emerged as a pivot in the development of cardiac arrhythmias as well as heart failure under various conditions and therefore represents a promising toehold for future therapies. Whereas the direct contribution of PKA to the development of an increased SR Ca^2+^ leak is still controversial, the crucial role of CaMKII in this context seems to be more established. There are convincing data from animal models as well as human tissue showing that an increased CaMKII activity in cardiac pathologies contributes to the manifestation of pump failure and arrhythmias and that CaMKII inhibition can yield beneficial effects. Thus, to our current knowledge, the diastolic SR Ca^2+^ leak can be addressed by two different approaches: direct stabilisation of the leaky RyR2 via suitable new compounds (RyR stabilisers) on the one hand and reduction of its hyperphosphorylation by CaMKII inhibition on the other hand. The clinical implications for both approaches would be various, stretching from arrhythmias to systolic pump failure as well as diastolic dysfunction.
